# Association between admission systolic blood pressure and major adverse cardiovascular events in patients with acute myocardial infarction

**DOI:** 10.1371/journal.pone.0234935

**Published:** 2020-06-19

**Authors:** Junyu Pei, Xiaopu Wang, Zhenhua Xing, Pengfei Chen, Wen Su, Simin Deng, Xinqun Hu

**Affiliations:** Department of Cardiovascular Medicine, The Second Xiangya Hospital, Central South University, Changsha, Hu'nan, China; Scuola Superiore Sant'Anna, ITALY

## Abstract

**Background:**

Several studies have previously demonstrated that higher systolic blood pressure level means lower risk of adverse cardiovascular outcomes. However, there is a lack of further investigation into the nonlinear relationship between admission systolic blood pressure (SBP) and adverse outcomes of acute myocardial infarction (AMI) patients.

**Objectives:**

The aim of this study was to investigate the specific relationship between admission SBP and incidence of major adverse cardiovascular events (MACE) in 30 days for AMI patients.

**Methods and results:**

Using data from the ACS-QUIK trial, we analyzed 21,364 patients from Kerala, India. In univariate linear-regression model, the OR was 0.90 per 10mmHg, the confidence interval (CI) was 95% (0.87–0.92) and *P* < 0.0001. The generalized additive model (GAM) showed a nearly U-shaped curve between admission SBP and MACE. Using a two-piecewise linear regression model, we calculated an inflection point of 159 mmHg. We found that the higher admission SBP is associated with lower incidence of MACE of AMI patients. In addition, subgroups with different LVEF have distinct effects on blood pressure-related outcomes. Lower SBP has a greater risk when LVEF < 40%.

**Conclusion:**

The present study revealed the U-shaped relationship between admission SBP and the risk of adverse cardiovascular outcome. The admission SBP could be a marker to provide clinical assessment and treatment.

**Trial registration:**

URL: http://www.clinicaltrials.gov. Unique identifier: NCT02256657

## Introduction

As a severe subset of coronary heart disease (CHD), acute myocardial infarction is still the most common cause of death in CHD patients. [1] Epidemiological data show that more than 780,000 people experience ACS each year in the United States, 70% of them will have NSTEMI. [2, 3] In high-income countries, the decline of mortality rates has been observed in patients with AMI since 1970s. [4,5] However, 80% of the world’s CHD deaths occurred in low and middle-income countries(LMIC), especially in South Asia. [6] In India, CHD is responsible for about 40% of the deaths in urban areas and 30% in rural areas. [7] Ameliorating prognosis, reducing mortality and promoting initial treatment have been key to the treatment of ACS in recent years.

In the Global Registry of Acute Coronary Events (GRACE) risk prediction tool proposed by Granger et al, systolic blood pressure (SBP) was listed as one of nine factors that independently predicted death and the combined end point in the initial 6 months after admission. For every 20 mmHg reduction in SBP, the odds ratio (OR) for hospital mortality increases by 1.4. [8–11] Additionally, several studies have previously demonstrated the inverse link between increasing admission SBP and the risk of adverse cardiovascular outcome. [12–15] However, there is a lack of further investigation into the nonlinear relationship between admission SBP and adverse outcome of AMI patients.

We use the data from the Acute Coronary Syndrome Quality Improvement in Kerala (ACS-QUIK) study to explore the association between admission SBP and MACE in AMI patients. The ACS-QUIK study is a cluster randomized, stepped-wedge clinical trial to assess the implementation and effect of a locally-developed quality improvement toolkit on MACE for ACS patients in Kerala, India. [16]

## Materials and methods

### Data source and study population

We obtained the database of the Biologic Specimen and Data Repository Information Coordinating Center (BioLINCC; National Institutions of Health, Bethesda, Maryland, USA). The database, analytic methods is not available to other researchers for any purposes because of the agreement with the BioLINCC.

We used the data from the Acute Coronary Syndrome Quality Improvement in Kerala (ACS-QUIK) trial, the rationale and design of which have been published and the results published. [17] The ACS-QUIK study enrolled 21,374 patients who presented with or who were transferred for evaluation and management of either an non–ST-segment elevation myocardial infarction (NSTEMI) or ST-segment elevation myocardial infarction (STEMI) in Kerala, India. However, the use of a quality improvement intervention compared with usual care did not decrease a composite of 30-day major adverse cardiovascular events among this group of patients. The variables we used in the database file were as follows: cohort, intervention, age, male sex, STEMI, heartrate, SBP, weight, smoking or tobacco, hypertension, peripheral arterial disease (PAD), prior transient ischemic attack (TIA) or stroke, diabetes, creatine kinase isoenzymes in the heart (CK-MB), troponin, hemoglobin (Hb), serum creatinine (sCr), high-density lipoprotein cholesterol (HDL-C), low density lipoprotein cholesterol (LDL-C), triglycerides (TRIG), fasting glucose, Killip class, left-ventricular ejection fraction (LVEF) category (1: <40%; 2: ≥40%; 3: = unknown or not assessed), cardiac arrest, angiography, PCI, coronary-artery bypass graft surgery (CABG) and major adverse cardiovascular events (MACEs).

### Exposure variables and outcome

We excluded from our study 299 ACS-QUIK study patients whose SBP (289 patients) or MACE (10 patients) data were not available. We divided the remaining patients into five groups (<100 mmHg, 100–119 mmHg, 120–139 mmHg, 140–159 mmHg and ≥160 mmHg) based on admission SBP. The primary outcome was MACE, including death, reinfarction (defined by the Third Universal Definition of Myocardial Infarction), stroke, and major bleeding, within 30 days of admission.

### Statistical analysis

Categorical variables are expressed as percentages, while continuous variables are presented as means ± standard deviations (SDs) or medians, depending on whether data distribution was normal (as assessed by normal quantile–quantile [Q–Q] plots). We used 1-way analysis of variance (ANOVA) and the Kruskal–Wallis H test to determine statistical differences between the means of the continuous variables, according to distribution type. Categorical variables were compared using chi-square tests. We first conducted a univariate analysis to determine if MACE within 30 days was related to admission SBP, and then used a univariate linear-regression model to evaluate the relationship between admission SBP and MACE within 30 days. We have presented both the non-adjusted and multivariate adjusted models. Then we use a multivariate linear regression model to calculate adjusted OR. In accordance with the STrengthening the Reporting of OBservational studies in Epidemiology (STROBE) statement, we have listed the results of the unadjusted, minimally adjusted and fully adjusted models in this study. We adjusted for cohort, intervention, age and male sex in the minimally adjusted model; and for cohort, intervention, age, male sex, STEMI, heartrate, weight, smoking or tobacco, hypertension, PAD, prior TIA or stroke, diabetes, cardiac arrest, Killip class, LVEF category, angiography, PCI, CABG and sCr level in the fully adjusted model. In addition, we used a generalized additive model (GAM) to determine whether admission SBP and MACE within 30 days had a nonlinear relationship. If we observed a nonlinear relationship, we used a 2-stage linear regression model to calculate the threshold effect of admission SBP on MACE within 30 days through a smooth curve. We then conducted a log-likelihood ratio analysis comparing the 1-line linear regression model with the 2-piecewise linear model. When the ratio of MACE to admission SBP was obvious in the smooth curve, the recursive method automatically calculated the inflection point by the maximum-likelihood model. All analyses were performed with the statistical-software packages R (The R Foundation; http://www.R-project.org) and EmpowerStats (X&Y Solutions, Inc., Boston, Massachusetts, US; http://www.empowerstats.com). *P*-values < 0.05 (two-sided) were considered statistically significant.

### Subgroup analysis

We used stratified linear-regression models to perform subgroup analysis and the likelihood ratio test to examine changes to each subgroup and interactions between subgroups. We divide the continuous variables from small to large into three groups and convert them into categorical variables. Then in examining the interaction term in each variable, we included the main effect and the interaction term.

## Results

### Baseline characteristics of participants

This study included 21,075 patients. All baseline characteristics are listed in Table 1. The average age of all participants was 60.11 ± 12 years; 75.71% of them were male. Participants with higher admission SBP had higher heart rate. In the high–admission SBP subgroup, participants had higher levels of troponin and Hb; fewer of them smoked; and they had relatively high levels of blood lipids such as HDL, LDL or TRIG. Serum creatinine levels did not differ significantly between groups.

**Table 1:** Baseline characteristics of participants.

SBP group	<100 mmHg	100–119 mmHg	120–139 mmHg	140–159 mmHg	≥160 mmHg	*P*-value
N	1056	3362	6326	5208	5412	
Intervention	509 (48.20%)	1720 (51.16%)	3366 (53.21%)	2829 (54.32%)	2879 (53.20%)	<0.001
Sex						<0.001
Female	260 (24.62%)	724 (21.53%)	1397 (22.08%)	1263 (24.25%)	1545 (28.55%)	
Male	796 (75.38%)	2638 (78.47%)	4929 (77.92%)	3945 (75.75%)	3867 (71.45%)	
Age	60.92 ± 12.42	59.31 ± 12.03	58.97 ± 11.96	59.78 ± 11.96	62.09 ± 11.77	<0.001
Heartrate (BPM)	75.38 ± 29.31	76.11 ± 18.44	77.29 ± 15.86	80.93 ± 17.64	85.32 ± 19.67	<0.001
SBP (mmHg)	82.84 ± 10.43	107.36 ± 5.12	126.57 ± 5.09	144.56 ± 5.14	176.98 ± 18.25	<0.001
Weight (Kg)	62.26 ± 9.13	63.16 ± 9.39	63.70 ± 9.36	63.66 ± 9.80	63.34 ± 10.32	<0.001
CK-MB (units/L)	72.71 ± 95.21	79.32 ± 92.28	96.76 ± 98.47	62.84 ± 89.77	67.30 ± 89.53	<0.001
Troponin (ng/ml)	13.17 ± 29.42	10.00 ± 23.14	9.25 ± 24.27	9.24 ± 24.74	7.71 ± 20.87	<0.001
Hb (g/dL)	12.62 ± 2.03	12.98 ± 2.00	13.17 ± 1.91	13.35 ± 1.99	13.36 ± 2.10	<0.001
Serum creatinine (units/L)	1.30 ± 0.59	1.16 ± 0.61	1.11 ± 0.60	1.14 ± 0.57	1.24 ± 0.81	<0.001
HDL-C (mg/dL)	40.56 ± 11.29	40.91 ± 10.60	41.24 ± 10.95	41.97 ± 10.36	43.02 ± 11.39	<0.001
LDL-C(mg/dL)	116.84 ± 41.98	115.85 ± 38.11	120.93 ± 40.14	125.17 ± 41.04	126.36 ± 42.22	<0.001
TRIG (mg/dL)	129.84 ± 74.50	129.64 ± 63.24	134.90 ± 70.01	139.73 ± 72.18	142.27 ± 75.71	<0.001
Fasting glucose (mg/dL)	158.90 ± 78.63	143.36 ± 69.10	143.69 ± 65.23	146.67 ± 66.11	154.55 ± 72.06	<0.001
Risk factors						
Smoking or tobacco	380 (35.98%)	1215 (36.14%)	2080 (32.88%)	1538 (29.53%)	1396 (25.79%)	<0.001
Hypertension	385 (36.46%)	1144 (34.03%)	2211 (34.95%)	2679 (51.44%)	3616 (66.81%)	<0.001
PAD	14 (1.33%)	32 (0.95%)	47 (0.74%)	48 (0.92%)	70 (1.29%)	0.032
Prior TIA or stroke	30 (2.84%)	76 (2.26%)	105 (1.66%)	104 (2.00%)	154 (2.85%)	<0.001
Diabetes	484 (45.83%)	1321 (39.29%)	2634 (41.64%)	2397 (46.03%)	2645 (48.87%)	<0.001
Cardiac state						
STEMI	826 (78.22%)	2343 (69.69%)	4334 (68.51%)	3160 (60.68%)	3017 (55.75%)	<0.001
Killip class						<0.001
1	645 (61.08%)	2921 (86.88%)	5742 (90.77%)	4618 (88.67%)	4528 (83.67%)	
2	92 (8.71%)	215 (6.40%)	306 (4.84%)	277 (5.32%)	293 (5.41%)	
3	55 (5.21%)	137 (4.07%)	222 (3.51%)	263 (5.05%)	561 (10.37%)	
4	264 (25.00%)	89 (2.65%)	56 (0.89%)	50 (0.96%)	30 (0.55%)	
LVEF category						<0.001
1	242 (22.92%)	486 (14.46%)	797 (12.60%)	661 (12.69%)	723 (13.36%)	
2	601 (56.92%)	2438 (72.51%)	4849 (76.65%)	3928 (75.42%)	4088 (75.54%)	
3	213 (20.17%)	438 (13.03%)	680 (10.75%)	619 (11.89%)	601 (11.10%)	
Cardiac arrest	88 (8.33%)	47 (1.40%)	58 (0.92%)	65 (1.25%)	50 (0.92%)	<0.001
Procedures						
Angiography	599 (56.72%)	1920 (57.11%)	3869 (61.16%)	3116 (59.83%)	3172 (58.61%)	<0.001
PCI	525 (49.72%)	1627 (48.39%)	3472 (54.88%)	2468 (47.39%)	2456 (45.38%)	<0.001
CABG	7 (0.66%)	19 (0.57%)	24 (0.38%)	28 (0.54%)	29 (0.54%)	0.561
MACE	227 (21.91%)	247 (7.46%)	293 (4.69%)	230 (4.47%)	244 (4.57%)	<0.001

SBP: systolic blood pressure. BPM: beats per minute. CK-MB: creatine kinase isoenzymes in the heart. Hb: hemoglobin. HDL-C: high-density lipoprotein cholesterol. LDL-C: low-density lipoprotein cholesterol. TRIG: triglycerides. PAD: peripheral arterial disease. TIA: transient ischemic attack. STEMI: ST-segment elevation myocardial infarction. PCI: percutaneous coronary intervention. LVEF: left-ventricular ejection fraction. LVEF category: 1: ≤40%; 2: >40% 3: = unknown or not assessed. CABG: coronary-artery bypass graft surgery. MACE: major adverse cardiovascular events.

### Relationship between admission SBP and MACE within 30 days

We used the univariate and multivariate linear-regression model to evaluate the relationship between admission SBP and MACE within 30 days. The non-adjusted model, minimally adjusted (Adjusted I) model and fully adjusted (Adjusted II) model are presented in Table 2. In the non-adjusted model, higher admission SBP was associated with lower rates of MACE (OR: 0.85; 95% confidence interval [CI], 0.84–0.87; *P* < 0.0001) per 10 mmHg. In the Adjusted I model (adjusted for cohort, intervention, age and male sex), there was no obvious change in results after adjustment (OR: 0.84; 95% CI, 0.83–0.86; *P* < 0.0001). The results of the Adjusted II model were OR: 0.90; 95% CI, 0.87–0.92; *P* < 0.0001 (Table 2). From the OR stratified by SBP bands, in these three groups (120–139, 140–159, >160), the OR value did not change significantly. We could find that the relationship between admission SBP and MACE is nonlinear.

**Table 2:** Relationship between SBP and MACE in different models.

Exposure	Non-adjusted	Adjusted I	Adjusted II
SBP per 10 mmHg	0.85 (0.84, 0.87), *P* < 0.0001	0.84 (0.83, 0.86), *P* < 0.0001	0.90 (0.87, 0.92), *P* < 0.0001
SBP group CO	0.69 (0.66, 0.73), *P* < 0.0001	0.67 (0.64, 0.71), *P* < 0.0001	0.76 (0.71, 0.82), *P* < 0.0001
SBP group (mmHg):			
<100	1.0	1.0	1.0
100–119	0.29 (0.24, 0.35), *P* < 0.0001	0.31 (0.25, 0.38), *P* < 0.0001	0.68 (0.49, 0.94), *P* = 0.0204
120–139	0.18 (0.15, 0.21), *P* < 0.0001	0.19 (0.16, 0.24), *P* < 0.0001	0.46 (0.33, 0.64), *P* < 0.0001
140–159	0.17 (0.14, 0.20), *P* < 0.0001	0.17 (0.14, 0.21), *P* < 0.0001	0.41 (0.29, 0.57), *P* < 0.0001
≥160	0.17 (0.14, 0.21), *P* < 0.0001	0.15 (0.13, 0.19), *P* < 0.0001	0.31 (0.22, 0.44), *P* < 0.0001

SBP: systolic blood pressure. MACE: major adverse cardiovascular events. CO: For CO, we converted the SBP group into a continuous variable. In the non-adjusted model, we did not adjust other covariates. In the Adjusted I model, we adjusted for cohort, intervention, age and male sex. In the Adjusted II model, we adjusted for cohort, intervention, age, male sex, ST-segment elevation myocardial infarction (STEMI), heartrate, weight, smoking or tobacco, hypertension, peripheral arterial disease (PAD), prior transient ischemic attack (TIA) or stroke, diabetes, cardiac arrest, Killip class, left-ventricular ejection fraction (LVEF) category, angiography, percutaneous coronary intervention (PCI), coronary-artery bypass graft surgery (CABG) and serum creatinine (sCr) level.

### Analyses of non-linear relationship

**Fig 1:**
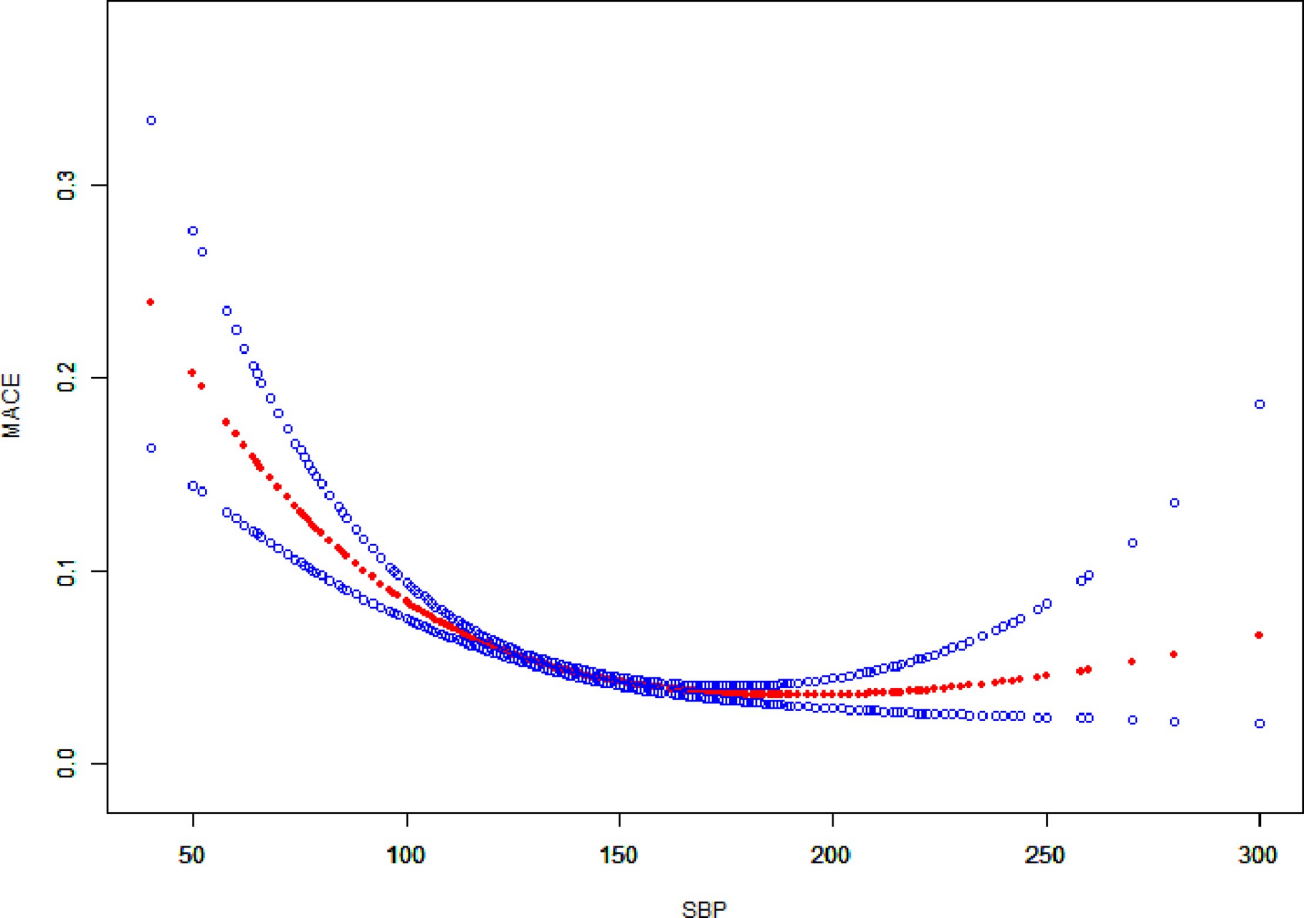
Relationship between admission Systolic Blood Pressure (SBP) and major adverse cardiovascular events (MACE). We adjusted for cohort, intervention, age, male sex, ST-segment elevation myocardial infarction (STEMI), heartrate, weight, smoking or tobacco, hypertension, peripheral arterial disease (PAD), prior transient ischemic attack (TIA) or stroke, diabetes, cardiac arrest, Killip class, left-ventricular ejection fraction (LVEF) category, angiography, percutaneous coronary intervention (PCI), coronary-artery bypass graft surgery (CABG) and serum creatinine (sCr) level.

Since admission SBP is a continuous variable, it is necessary to study the nonlinear relationship between it and MACE within 30 days. The GAM showed a U-shaped curve between these 2 variables (Fig 1), and the R^2^ was 0.1414. Using a 2-piecewise linear-regression model, we calculated the inflection point as 159 mmHg (P for trend< 0.001). MACE on the left side of the inflection point showed a downward trend, whereas on the right side there was an adverse upward trend. However, there was no statistical association between changes in admission SBP and MACE on the right side of the inflection point (Table 3).

**Table 3:** Results of two-piecewise linear-regression model.

Outcome: MACE			
Exposure: SBP			
LVEF category	1	2	Total
OR of Linear-regression model per 10 mmHg	0.82 (0.79, 0.86), *P* < 0.0001	0.92 (0.89, 0.95), *P* < 0.0001	0.90 (0.87, 0.92), *P* < 0.0001
Inflection point (K)	148	159	159
<K Effect size OR (95%CI)	0.76 (0.71, 0.82), *P* < 0.0001	0.86 (0.82, 0.91), *P* < 0.0001	0.82 (0.79, 0.85), *P* < 0.0001
>K Effect size OR (95%CI)	1.00 (0.89, 1.13), *P* = 0.9731	1.06 (0.97, 1.16), *P* = 0.2192	1.08 (1.00, 1.16), *P* = 0.0367
P for trend	0.002	0.002	<0.001

MACE: major adverse cardiovascular events. SBP: systolic blood pressure. Adjusted for cohort, intervention, age, male sex, ST-segment elevation myocardial infarction (STEMI), heartrate, weight, smoking or tobacco, hypertension, peripheral arterial disease (PAD), prior transient ischemic attack (TIA) or stroke, diabetes, cardiac arrest, Killip class, left-ventricular ejection fraction (LVEF) category, LVEF category: 1: <40%; 2: ≥40%; 3: = unknown or not assessed, angiography, percutaneous coronary intervention (PCI), coronary-artery bypass graft surgery (CABG) and serum creatinine (sCr) level.

### Subgroup analysis and interaction test

**Fig 2:**
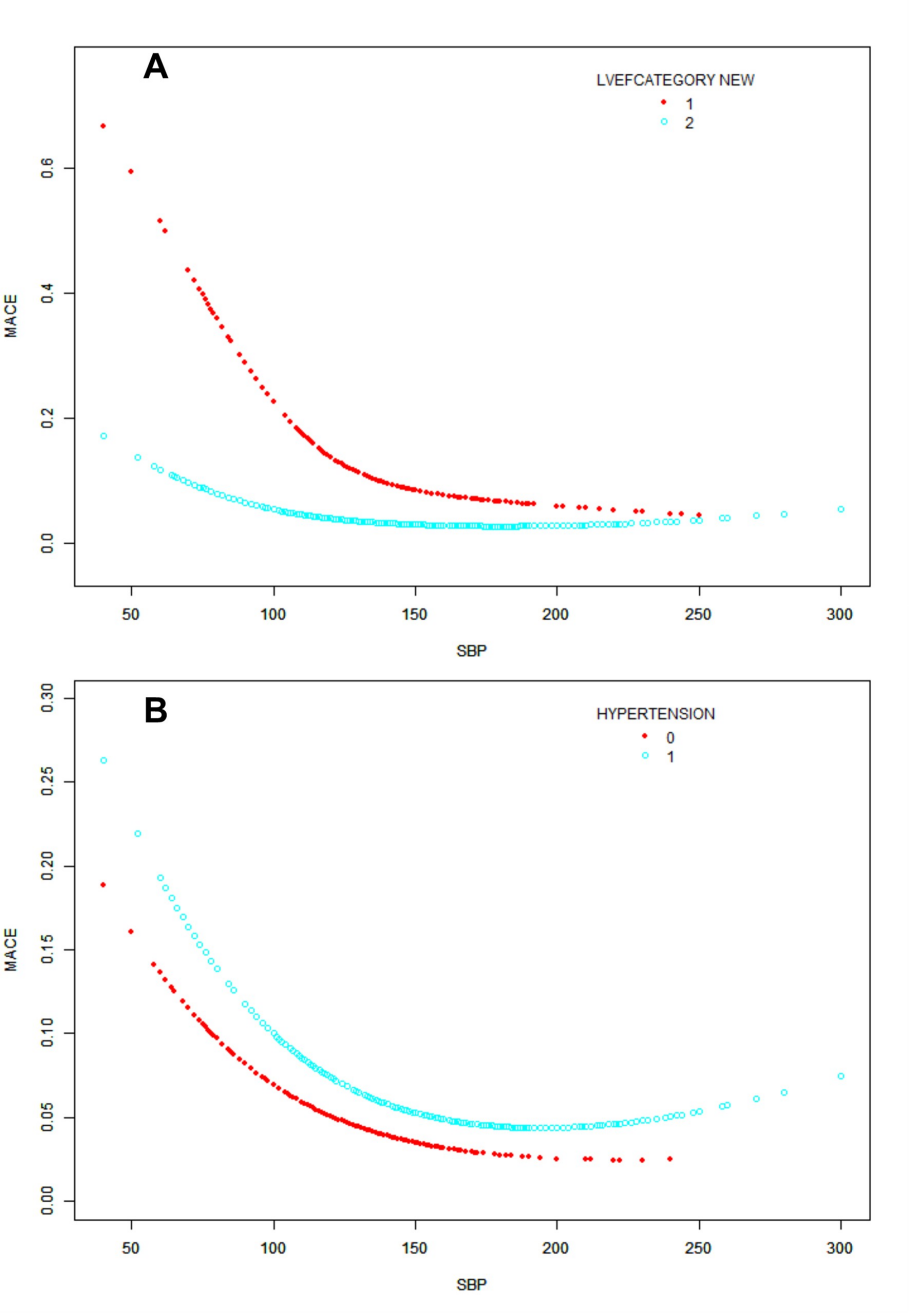
(A) Relationship between admission SBP and MACE after grouping by left-ventricular ejection fraction (LVEF). LVEF category: 1: <40%; 2: ≥40%; 3: = unknown or not assessed (B) Relationship between admission SBP and MACE after grouping by prior hypertension.

Results of the stratified analysis are shown in S1 Table. The interaction test showed statistical significance for LVEF category (*P* = 0.0006), based on which we therefore performed a stratified analysis. We found that the OR of LVEF < 40% are less than LVEF ≥ 40% (0.82 vs 0.92), and their 95%CI are not overlapped (0.79–0.86; P< 0.0001 vs 0.89–0.95; P< 0.0001) (Table 3). In those with LVEF,40%, low SBP means higher risk than patients with LVEF ≥ 40% (Fig 2).There was no significant change in the relationship between SBP and MACE, regardless of whether the patient had previous hypertension (Fig 2).

## Discussion

In this study of 21,075 patients from the ACS-QUIK trial, we found that admission SBP formed a nearly U-shaped curve with the incidence of MACE within 30 days, and that the inflection point was 159 mmHg. When the admission SBP was <159 mmHg, the incidence of MACE decreased by 15% for every additional 10 mmHg of admission SBP. Therefore, higher admission SBP (SBP < 159mmHg) was associated with lower incidence of MACE in AMI patients.

Blood pressure is one of the most readily available physiological indices that can be obtained within the first few minutes of AMI patients entering the emergency room, and it is even more easily obtained than other clinical data such as troponin level. Therefore, admission SBP can be used as a predictive factor for fast clinical evaluation and adverse cardiovascular outcome studies. If it can be thus used effectively to assess the risk of adverse outcome, then a treatment plan for new AMI patients may be quickly drawn up.

Our conclusions essentially proved the studies we mentioned previously. In the Acute Coronary Syndrome Israel Survey (ACSIS) study, which included 7645 patients diagnosed with MI, the findings revealed that SBP is related to cardiovascular events and total mortality. Patients with SBP <110 mm Hg displayed significantly increased hazard ratios (HR) for 7‐day, 1‐year mortality and MACE of 2.37, 1.92 and 1.51 compared to those who have normal admission SBP(110mmHg-140mmHg). [13] The results of a prospective multicenter observational study on 11,292 Korean patients with STEMI found that those with normal SBP (100–139 mmHg) had a higher risk of in-hospital mortality compared with hypertension patients, but not of all‐cause death or MACE. [15] Another study on 3943 patients with acute MI reached a similar conclusion [12]. In addition, using data from more than 10,000 non–ST‐elevation ACS patients in two large registries, Lee et al. found an independent relationship between lower SBP and in‐hospital mortality; hypertension history or use of antihypertensive medication did not affect these associations [14]. Nevertheless, only a few studies have examined whether admission SBP levels *per se* might affect hard outcomes in AMI patients [18]. Previous studies have been limited to the association between higher admission SBP and better outcome, so that the specific relationship between admission SBP and adverse outcome is not clearly clarified. Our study revealed a nonlinear relationship between admission SBP and MACE within 30 days, calculated admission SBP’s specific relationship curve with MACE and obtained an inflection point. Subsequently, we also performed a subgroup analysis based on different LVEFs and obtained the relationship of admission SBP and changes thereto to prognosis under different cardiac-function conditions. Our findings might reveal the effect of admission SBP on short-term outcomes in patients with AMI complicated by heart failure, and we hope it will provide some new tips for clinical assessment and treatment.

As the findings of the study by Boersma et al. using the Platelet Glycoprotein IIb–IIIa in Unstable Angina: Receptor Suppression Using Integrilin Therapy (PURSUIT) trial database, [19] low SBP has a certain effect on all-cause mortality in patients with MI. As an indicator of poor cardiac reserve, low SBP always presents in patients with severe heart failure and cardiogenic shock. [20] It indicates insufficient perfusion of tissue and coronary arteries, which further exacerbates the burden of coronary ischemia in patients with MI. This explains why lower SBP has a greater risk when LVEF < 40% in our study. Our research shows that subgroups with different LVEF have distinct effects on blood pressure-related outcomes. The OR value of LVEF < 40% is less than LVEF ≥ 40% (0.82 vs 0.92), and in subgroup that LVEF < 40%, the curve of patients with low SBP decreased steeper (Fig 2). LVEF below 40% means, in many ways, a decrease in cardiac function, and existing analysis shows that mortality due to heart failure during ACS hospitalization is very high [21]. Putte et al. and GRACE registration studies have shown that ACS events accompanied by heart failure increased the risk of MACE events within 30 days. The ONTARGET and TRANSCEND trials has also drew similar results that a U-shaped curve occurred with a particular greater risk at lower SBP and DBP in heart failure patients. [22] The increase of coronary artery perfusion is accompanied by SBP rising, for which can explain the downward trend of MACE with the rising of admission SBP. There are also some studies thought that this effect may associate with the increasing comorbidities and frailty in myocardial infraction (MI) patients with heart failure. [23] In addition, we hypothesize that with the SBP rising, as the blood flow rate increases, the possibility of thrombosis is likely to be lower, which may affect the occurrence of MACE.

Our finding showed that the inflection point is 159mmHg, then the curve presented an upward trend but was not statistically significant. We suspect that the sample size was not large enough or that there were other errors. As sample size increases, we believe that there will be different changes to the curve after the inflection point (159 mmHg), because abnormal vascular reactivity and the complex interaction of vascular-wall shear stress with neurohormone activation, both caused by hypertension, trigger the development of endothelial dysfunction, vascular-wall remodeling and atherosclerotic lesions. [24–26] Coronary-plaque rupture eventually leads to ACS in the high-coagulation state.

It is worth mentioning that patients with prior hypertension had a higher incidence of MACE compared with those without. After adjusting the demographic data, we found no statistically significant difference in admission SBP. This suggested that hypertension could have a negative effect on the prognosis of ACS, which is consistent with current findings on the pathophysiological mechanisms of interaction between hypertension and ACS. [27] However, there are no data on the use of antihypertensive drugs in this scenario, and therefore, we cannot rule out the effects of such drugs.

In general, we believe that clinical research into pathophysiological mechanisms in admission SBP and the prognosis of ACS patients need further development in order to provide effective advice for the diagnosis and treatment of ACS patients in clinical practice.

## Limitations

Some values were missing from BioLINCC database data; also, several variables in the GRACE score, such as excessive deletion of CK-MB, were not included. We could adjust only for the variables that existed in the database, and we have done so as much as possible. In addition, some patients refused coronary angiography or PCI, resulting in a lower rate of reperfusion with primary angioplasty of patients, which may affect the results of the study. Also, note that the population included in the ACS-QUIK trial was of Asian ethnicity, and we did not adjust for the regional differences in this population. Moreover, they were all from developing countries, in which there might be some restrictions on the quality and concept of diagnosis and treatment.

## Conclusion

The present study revealed the U-shaped relationship between admission SBP and the risk of adverse cardiovascular outcome. The admission SBP could be a marker to provide clinical assessment and treatment.

## Supporting information

S1 Table Subgroup analysis and interaction test. (DOCX)
