# Supplementary material for: Association between admission systolic blood pressure and major adverse cardiovascular events in patients with acute myocardial infarction
Source: PLoS One. 2020 Jun 19;15(6):e0234935. doi: 10.1371/journal.pone.0234935 (PMC7304596; doi:10.1371/journal.pone.0234935)
Supplement: S1 Table — (DOCX) [file pone.0234935.s001.docx]

**S1 Table. Subgroup analysis and interaction test.**

|  | N | OR (95% CI) | *P*-value | *P* for interaction |
| --- | --- | --- | --- | --- |
| Intervention |  |  |  | 0.4152 |
| No | 10,061 | 0.90 (0.86, 0.95) | *P* = 0.0002 |  |
| Yes | 11,303 | 0.90 (0.86, 0.93) | *P* < 0.0001 |  |
| Age |  |  |  | 0.4212 |
| Young | 6977 | 0.86 (0.80, 0.93) | *P* = 0.0002 |  |
| Middle | 6780 | 0.93 (0.88, 0.99) | *P* = 0.0176 |  |
| Old | 7607 | 0.89 (0.86, 0.93) | *P* < 0.0001 |  |
| Sex |  |  |  | 0.5085 |
| Female | 5189 | 0.89 (0.85, 0.94) | *P* < 0.0001 |  |
| Male | 16,175 | 0.90 (0.87, 0.93) | *P* < 0.0001 |  |
| STEMI |  |  |  | 0.7356 |
| No | 7684 | 0.92 (0.88, 0.97) | *P* = 0.0007 |  |
| Yes | 13,680 | 0.88 (0.84, 0.91) | *P* < 0.0001 |  |
| Heart rate |  |  |  | 0.9519 |
| Low | 7062 | 0.87 (0.82, 0.93) | *P* < 0.0001 |  |
| Moderate | 6838 | 0.89 (0.83, 0.95) | *P* = 0.0003 |  |
| High | 7463 | 0.91 (0.87, 0.94) | *P* < 0.0001 |  |
| Weight |  |  |  | 0.126 |
| Low | 6893 | 0.87 (0.82, 0.91) | *P* < 0.0001 |  |
| Moderate | 7076 | 0.89 (0.85, 0.94) | *P* < 0.0001 |  |
| High | 7395 | 0.93 (0.88, 0.98) | *P* = 0.0103 |  |
| Smoking or tobacco | |  |  | 0.3447 |
| No | 14,755 | 0.90 (0.87, 0.94) | *P* < 0.0001 |  |
| Yes | 6609 | 0.89 (0.84, 0.94) | *P* < 0.0001 |  |
| Hypertension |  |  |  | 0.1896 |
| No | 11,329 | 0.87 (0.83, 0.92) | *P* < 0.0001 |  |
| Yes | 10,035 | 0.92 (0.88, 0.95) | *P* < 0.0001 |  |
| PAD |  |  |  | 0.9646 |
| No | 21,153 | 0.90 (0.87, 0.92) | *P* < 0.0001 |  |
| Yes | 211 | 0.96 (0.72, 1.28) | *P* = 0.7651 |  |
| Prior stroke or TIA |  |  |  | 0.0811 |
| No | 20,895 | 0.89 (0.87, 0.92) | *P* < 0.0001 |  |
| Yes | 469 | 0.94 (0.83, 1.07) | *P* = 0.3732 |  |
| Diabetes |  |  |  | 0.1827 |
| No | 11,883 | 0.91 (0.87, 0.95) | *P* < 0.0001 |  |
| Yes | 9481 | 0.89 (0.85, 0.92) | *P* < 0.0001 |  |
| Angiography |  |  |  | 0.4262 |
| No | 8688 | 0.89 (0.85, 0.92) | *P* < 0.0001 |  |
| Yes | 12,676 | 0.90 (0.86, 0.95) | *P* < 0.0001 |  |
| PCI |  |  |  | 0.2011 |
| No | 10,816 | 0.89 (0.86, 0.92) | *P* < 0.0001 |  |
| Yes | 10,548 | 0.90 (0.85, 0.95) | *P* = 0.0002 |  |
| Cardiac arrest |  |  |  | 0.1079 |
| No | 21,056 | 0.89 (0.86, 0.92) | *P* < 0.0001 |  |
| Yes | 308 | 0.99 (0.87, 1.13) | *P* = 0.8777 |  |
| CABG |  |  |  | 0.9811 |
| No | 21257 | 0.90 (0.87, 0.92) | *P* < 0.0001 |  |
| Yes | 107 | — |  |  |
| Killip class |  |  |  | 0.4149 |
| 1 | 18,454 | 0.89 (0.86, 0.93) | *P* < 0.0001 |  |
| 2 | 1183 | 0.91 (0.81, 1.01) | *P* = 0.0752 |  |
| 3 | 1238 | 0.91 (0.84, 0.97) | *P* = 0.0061 |  |
| 4 | 489 | 1.00 (0.88, 1.13) | *P* = 0.9788 |  |
| LVEF category |  |  |  | 0.0006 |
| 1 | 2864 | 0.82 (0.78, 0.86) | *P* < 0.0001 |  |
| 2 | 15,730 | 0.92 (0.89,0.96) | *P* < 0.0001 |  |
| 3 | 2481 | 0.84 (0.78,0.91) | *P* < 0.0001 |  |
| Creatinine level |  |  |  | 0.295 |
| Low | 3280 | 0.86 (0.80, 0.94) | *P* = 0.0005 |  |
| Moderate | 5786 | 0.95 (0.89, 1.00) | *P* = 0.0487 |  |
| High | 4765 | 0.88(0.85, 0.92) | *P* < 0.0001 |  |

We divide the continuous variables from small to large into three groups on average and be converted into categorical variables.

CI: confidence interval. STEMI: ST-segment elevation myocardial infarction. PAD: peripheral arterial disease. TIA: transient ischemic attack. PCI: percutaneous coronary intervention. CABG: coronary-artery bypass graft surgery. LVEF: left-ventricular ejection fraction. LVEF category: 1: ≤40%; 2: ＞40% 3: = unknown or not assessed.
